# Is physician assessment of alcohol consumption useful in predicting risk of severe liver disease among people with HIV and HIV/HCV co-infection?

**DOI:** 10.1186/s12889-019-7608-1

**Published:** 2019-10-15

**Authors:** Milensu Shanyinde, Enrico Girardi, Massimo Puoti, Andrea De Luca, Laura Sighinolfi, Uberti Foppa Caterina, Pietro Caramello, Fiona C. Lampe, Antonella D’Arminio Monforte, Alessandro Cozzi-Lepri, for the ICONA Foundation Study Group, A. d’Arminio Monforte, M. Andreoni, G. Angarano, A. Antinori, F. Castelli, R. Cauda, G. Di Perri, M. Galli, R. Iardino, G. Ippolito, A. Lazzarin, C. F. Perno, F. von Schloesser, P. Viale, A. Castagna, F. Ceccherini-Silberstein, A. Cozzi-Lepri, E. Girardi, S. Lo Caputo, C. Mussini, M. Puoti, A. Ammassari, C. Balotta, A. Bandera, P. Bonfanti, S. Bonora, M. Borderi, A. Calcagno, L. Calza, M. R. Capobianchi, A. Cingolani, P. Cinque, A. De Luca, A. Di Biagio, N. Gianotti, A. Gori, G. Guaraldi, G. Lapadula, M. Lichtner, G. Madeddu, F. Maggiolo, G. Marchetti, S. Marcotullio, L. Monno, S. Nozza, E. Quiros Roldan, R. Rossotti, S. Rusconi, M. M. Santoro, A. Saracino, M. Zaccarelli, I. Fanti, L. Galli, P. Lorenzini, A. Rodano, M. Shanyinde, A. Tavelli, F. Carletti, S. Carrara, A. Di Caro, S. Graziano, F. Petrone, G. Prota, S. Quartu, S. Truffa, A. Giacometti, A. Costantini, C. Valeriani, C. Santoro, C. Suardi, V. Donati, G. Verucchi, C. Minardi, T. Quirino, C. Abeli, P. E. Manconi, P. Piano, B. Cacopardo, B. Celesia, J. Vecchiet, K. Falasca, L. Sighinolfi, D. Segala, F. Mazzotta, F. Vichi, G. Cassola, C. Viscoli, A. Alessandrini, N. Bobbio, G. Mazzarello, C. Mastroianni, V. Belvisi, I. Caramma, A. Chiodera, P. Milini, G. Rizzardini, A. L. Ridolfo, R. Piolini, S. Salpietro, L. Carenzi, M. C. Moioli, C. Tincati, C. Puzzolante, N. Abrescia, A. Chirianni, G. Borgia, R. Orlando, F. Di Martino, L. Maddaloni, I. Gentile, G. Bonadies, A. Cascio, C. Colomba, F. Baldelli, E. Schiaroli, G. Parruti, T. Ursini, G. Magnani, M. A. Ursitti, V. Vullo, A. Cristaudo, G. Baldin, S. Cicalini, L. Gallo, E. Nicastri, R. Acinapura, M. Capozzi, R. Libertone, S. Savinelli, A. Latini, G. Iaiani, L. Fontanelli Sulekova, M. Cecchetto, F. Viviani, M. S. Mura, B. Rossetti, D. Francisci, C. Di Giuli, P. Caramello, G. C. Orofino, M. Sciandra, M. Bassetti, A. Londero, G. Pellizzer, V. Manfrin

**Affiliations:** 1 Institute of Global Health, University College London, Royal Free Hospital, London, UK; 2 Lazzaro Spallanzani National Institute for Infectious Diseases, Rome, Italy; 3 Niguarda ca Grande Hospital, Milan, Italy; 4 University Hospital of Siena, Siena, Italy; 5 Division of Infectious Diseases, Hospital of Ferrara, Ferrara, Italy; 6 Infectious disease university San Raffaele, San Raffaele, Italy; 7 Infectious and Tropical Diseases Unit I, Department of Infectious Diseases, Amedeo di Savoia Hospital, Torino, Italy; 8 Clinic of Infectious Diseases, San Paulo Hospital, Milan, Italy

**Keywords:** HIV-infected, HIV/HCV co-infection, Alcohol consumption, Severe liver disease

## Abstract

**Background:**

Alcohol consumption is a known risk factor for liver disease in HIV-infected populations. Therefore, knowledge of alcohol consumption behaviour and risk of disease progression associated with hazardous drinking are important in the overall management of HIV disease. We aimed at assessing the usefulness of routine data collected on alcohol consumption in predicting risk of severe liver disease (SLD) among people living with HIV (PLWHIV) with or without hepatitis C infection seen for routine clinical care in Italy.

**Methods:**

We included PLWHIV from two observational cohorts in Italy (ICONA and HepaICONA). Alcohol consumption was assessed by physician interview and categorized according to the National Institute for Food and Nutrition Italian guidelines into four categories: abstainer; moderate; hazardous and unknown. SLD was defined as presence of FIB4 > 3.25 or a clinical diagnosis of liver disease or liver-related death. Cox regression analysis was used to evaluate the association between level of alcohol consumption at baseline and risk of SLD.

**Results:**

Among 9542 included PLWHIV the distribution of alcohol consumption categories was: abstainers 3422 (36%), moderate drinkers 2279 (23%), hazardous drinkers 637 (7%) and unknown 3204 (34%). Compared to moderate drinkers, hazardous drinking was associated with higher risk of SLD (adjusted hazard ratio, aHR = 1.45; 95% CI: 1.03–2.03). After additionally controlling for mode of HIV transmission, HCV infection and smoking, the association was attenuated (aHR = 1.32; 95% CI: 0.94–1.85). There was no evidence that the association was stronger when restricting to the HIV/HCV co-infected population.

**Conclusions:**

Using a brief physician interview, we found evidence for an association between hazardous alcohol consumption and subsequent risk of SLD among PLWHIV, but this was not independent of HIV mode of transmission, HCV-infection and smoking. More efforts should be made to improve quality and validity of data on alcohol consumption in cohorts of HIV/HCV-infected individuals.

## Background

Identifying unhealthy levels of alcohol consumption in HIV patients seen for routine clinical care is important because of the possible role alcohol plays in HIV disease progression as well as non HIV-related comorbidities such as liver failure [1]. In the era of combination antiretroviral treatment (cART), people living with HIV/AIDS (PLWHIV) are now living longer and long term effects of alcohol consumption are likely to affect people’s quality of life and survival [2]. Therefore, knowledge of alcohol consumption behaviour and risk of disease progression associated with hazardous drinking are important in the overall management of HIV disease [3]. Alcohol consumption is common in PLWHIV with estimates of current alcohol use reported to be 50% in studies of HIV-positive people [1, 4–8] and hazardous drinking, has reported prevalence ranging between 8 and 12%. Hazardous drinking can lead to harmful consequences such as liver disease progression or liver related mortality [7, 9–18].

Assessment of alcohol consumption in HIV cohort studies varies because of the measurement tools implemented, mode of assessment and risk groups under investigation [18–25]*.* Most studies have used methods of alcohol assessment based on brief self-reported questionnaires relating to quantity and/or frequency of drinks consumed [26]. Others studies have used patient interviews, biomarkers or breath tests to assess level of alcohol consumption [27, 28]. These different measurement tools has led to methodological challenges in quantifying estimates of alcohol consumption amongst PLWHIV [29].

In this analysis, we use data routinely collected by treating physicians in two cohorts of PLWHIV seen for routine clinical care in Italy, including questions related to both frequency and quantity of alcohol consumed. Our objective is two-fold. Firstly, we aim to categorise drinking behaviour using data routinely collected in our cohorts by mapping the questions on the electronic case report form (CRF) to those used in national drinking guidelines known as the National Institute for Food and Nutrition (NIFN) in Italy. Secondly, to assess the association between alcohol consumption and risk of severe liver disease (SLD) among PLWHIV with or without HCV infection.

## Methods

### Study participants

This analysis includes all PLWHIV (with and without HCV co-infection) enrolled up to June 2016 in the ICONA Foundation Study and HepaICONA prospective cohorts who were free from SLD (see definition in paragraph below) at study enrolment. Patients enrolled prior to 1st January 2002 were excluded from this study as alcohol assessment in the cohorts was not in standard use prior to this date. Both cohorts are observational studies of PLWHIV and details of both cohorts have been published elsewhere [30, 31]. In brief, the ICONA Foundation cohort began to enrol PLWHIV in 1997 as long as they were antiretroviral (ART)-naïve at time of enrolment. Patients’ demographics, clinical and laboratory data are recorded using an electronic data collection form. Occurrence of any clinical event, including liver-related events and causes of death (classified using CoDe) are recorded [30]. The HepaICONA cohort began in 2013 and it enrols HIV/HCV co-infected and HCV viremic individuals who are naive to direct acting antivirals drugs (DAA) at study entry. Similar data collections processes are implemented as those in place for the ICONA cohort, including the questions related to alcohol consumptions [31]. All patients have given informed consent to participate in the study and ethic committee approval from all participating centres was obtained for both cohorts (Additional file 1: Table S1).

### Classification of alcohol consumption

Alcohol consumption is collected by physician interview at study enrolment and at subsequent clinical visits (at least every 6 months) during follow-up. This analysis only includes assessments carried out at enrolment (baseline), which for the ICONA cohort is prior to ART initiation. Exact questions in the patients’ interview (with possible responses) are as follows; 1) Do you currently drink alcohol? (Yes/No/Unknown); 2) How frequently do you drink alcohol? (Daily/Non-daily/Unknown); 3) How many units of (Wine/Beer/Spirits) do you consume per day?

Frequency and quantity of units of drink consumed was translated into drinking categories by mapping the data to the definitions described in the NIFN guidelines. A unit of alcohol in Italy is defined as containing 14 g of pure alcohol which corresponds to 125 ml of wine, 330 ml of a can of beer and 40 ml of liquor [32].

Hazardous drinking is defined as > 3 units/day for men and for women > 2 units/day. In cases where drinking was reported as ‘non-daily’ WHO guidelines were used which state that > 4 drinks per occasion is considered hazardous drinking [33]. People were classified as moderate drinkers if they consumed an amount below the hazardous drinking thresholds. Abstainers were people who reported not drinking alcohol at all.

**Fig. 1:**
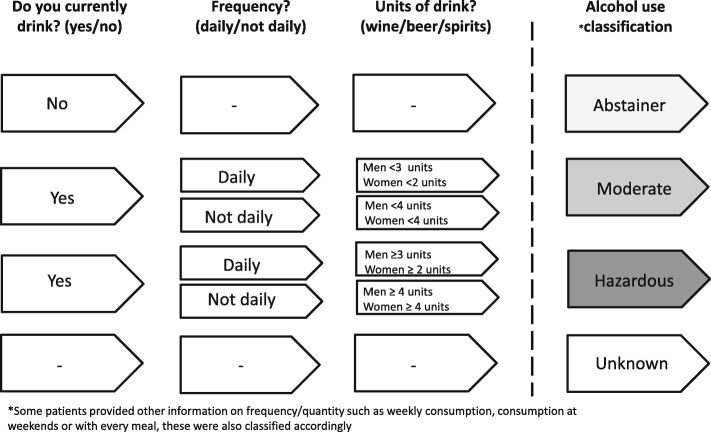
Alcohol classification based on responses from Physician assessment of alcohol consumption

Alcohol consumption at baseline was categorised into these three groups described above and an unknown category if there was missing data for alcohol consumption as shown in Fig. 1. In some individuals who reported more than one type of drink, the drink with the highest quantity of alcohol was used in the classification process. In instances were information on alcohol was given in other metrics e.g. ml, this was converted to units (e.g. 500 ml of wine a day equated to 500 ml/125 ml = 4 units/day). In few instances were information reported as having a drink with a meal, this was assumed 2 units/day to take into account of the 2 meals a day.

### Definitions of covariates

Demographics were collected at study enrolment. HIV-related variables (previous AIDS diagnosis, CD4 and HIV-RNA), hepatitis co-infection (HCV) status and smoking status were also collected at study enrolment. HCV status was based on HCVAb+ test or, when the results for antibody test was not available, using HCV-RNA > 615 IU/mL or a positive HCV-RNA qualitative test or evidence of the determination of HCV genotype.

### Definition of severe liver disease

Time to the development of severe liver disease in follow-up was a composite endpoint defined at the time of experiencing one of the following events (first of these occurring): (i) a FIB4 > 3.25 (where FIB4 was calculated based on available blood test results using the formula (age*AST)/PLT*sqrt (ALT) and assessed at each clinical visit [34];(ii) a clinical diagnosis of liver disease from medical records (ascites, decompensated cirrhosis, hepatocellular carcinoma, hepatic encephalopathy, oesophageal varices); (iii) liver-related death.

### Statistical analyses

Baseline was defined as date of enrolment between 1st January 2002 and 30th June 2016 in ICONA and between 1st October 2013 and 30th June 2016 for people in HepaICONA. Individuals were followed up until the date of experiencing the composite endpoint or their follow-up was censored at the date of their clinical visit at which they were last seen free from SLD. Summary statistics were used to describe the study participants overall and after stratification by alcohol consumption category at baseline. Formal comparisons of categorical characteristics by groups of alcohol consumption were performed using chi-squared tests.

Time to SLD was estimated using the Kaplan Meier method overall and after stratifying by baseline alcohol consumption category. Univariable and multivariable Cox regression models were fitted to estimate hazard ratios (HRs) of the risk of SLD associated with baseline levels of alcohol consumption. In the Cox regression model, only time-fixed covariates measured at baseline were included. Potential confounders measured at baseline were considered in a series of separate multivariable models fitted sequentially as follows: Model #1 controlling for demographic factors (gender, age, nationality, geographical region, calendar year of enrolment); Model #2 (model #1 plus previous AIDS diagnosis, CD4, HIV-RNA, and HBV infection status); Model #3 (model #2 plus mode of HIV transmission, and HCV infection status) and finally model #4 (model #3 plus smoking status). Results are presented as adjusted hazard ratios (aHRs) with 95% confidence intervals (95% CI). An interaction term between HCV infection and alcohol consumption was formally tested to determine whether the effect of alcohol consumption on risk of SLD differed by HCV infection status. This could be done only for the ICONA cohort as HepaICONA includes only HIV/HCV co-infected individuals. Two different methods for handling missing data on alcohol consumption were applied: (i) people with unknown alcohol consumption were included as a separate ‘unknown’ category of the alcohol variable (main analysis); (ii) an analysis in which people with unknown information were re-classified as ‘Abstainer’, ‘Moderate’ or ‘Hazardous’ drinker using multiple imputation (MI). A Fully Conditional Specification (FCS) imputation algorithm was implemented owing to the discrete nature of the alcohol consumption variable. We assumed 10 imputes with 100 iterations to be sufficient. Variables considered predictors of unreported alcohol consumption included in the MI model were: age, gender, nationality, geographical region, calendar year enrolled, mode of HIV transmission, AIDS diagnosis, CD4, HIV-RNA, HCV infection, smoking status and SLD. Separate univariable and multivariable Cox models were fitted similar to those used for the main analysis. Using Rubin’s combination rules, which is a method that combines estimates from imputed datasets to estimate standard errors, confidence intervals, and *p*-values to give an overall estimate of the imputed datasets. All analyses were performed using SAS (version 9.4, SAS Institute, Cary North Carolina USA).

## Results

### Alcohol consumption

We included 9542 patients who satisfied the inclusion criteria (8876 from ICONA and 666 from HepaICONA). When mapping our questions to the NIFN guidelines the distribution according to alcohol consumption was; abstainers 3422 (36%; 95%CI [35–37]), moderate users 2279 (23%; 95%CI [23–25]), hazardous drinkers 637 (7%; 95%CI [6, 7]), and unknown alcohol status 3204 (34%; 95%CI [33–35]). The distribution according to alcohol consumption in individuals with available data on alcohol consumption (*n* = 6338) was; abstainers (54%; 95%CI [53–55]), moderate users (36%; 95%CI [35–37]) and hazardous drinkers (10%; 95%CI [9–11]). After using MI to reclassify people with missing data, overall distribution of alcohol consumption was as follows: abstainers (53%), moderate users (37%) and hazardous drinkers (10%).

### Patient characteristics

Table 1 shows baseline characteristics for (*n* = 6338), overall and stratified by alcohol consumption. The majority of individuals were male (77%); median age [IQR] 38 [31–46] years. Compared to moderate drinkers, hazardous drinkers were more likely to be male (*p* < 0.001), of older age (*p* < 0.001), injecting drug users (*p* < 0.001), smokers (*p* < 0.001) and HIV/HCV co-infected (*p* < 0.001). Compared to individuals with complete data on alcohol consumption, those with missing data were more likely to be male (*p* < 0.001), of older age (*p* < 0.001), to have acquired HIV through IDU (*p* < 0.001), to not report smoking status (*p* < 0.001), to have no test result for HCV infection (*p* < 0.001) and to be enrolled in later calendar years (*p* < 0.001) (Additional file 2: Table S2).

**Table 1:** Patients’ characteristics stratified by alcohol consumption classification at baseline

Baseline characteristics	Abstainers (*N* = 3422)	Moderate (*N* = 2279)	Hazardous (*N* = 637)	Total (*N* = 6338)
Gender, n(%)				
Male	2363 (69.1%)	1954 (85.7%)	567 (89.0%)	4884 (77.1%)
Female	1059 (30.9%)	325 (14.3%)	70 (11.0%)	1454 (22.9%)
Age, years				
Median (IQR)	38 (31, 47)	37 (30, 45)	41 (34, 49)	38 (31, 46)
Mode of HIV Transmission, n(%)				
PWID	367 (10.7%)	250 (11.0%)	114 (17.9%)	731 (11.5%)
Homosexual contacts	1317 (38.5%)	1124 (49.3%)	222 (34.9%)	2663 (42.0%)
Heterosexual contacts	1517 (44.3%)	757 (33.2%)	269 (42.2%)	2543 (40.1%)
Other/Unknown	221 (6.5%)	148 (6.5%)	32 (5.0%)	401 (6.3%)
Nationality, n(%)				
Italian	2572 (75.2%)	1915 (84.0%)	516 (81.0%)	5003 (78.9%)
Region, n(%)				
North	1577 (46.1%)	1144 (50.2%)	382 (60.0%)	3103 (49.0%)
Center	1366 (39.9%)	866 (38.0%)	217 (34.1%)	2449 (38.6%)
South	479 (14.0%)	269 (11.8%)	38 (6.0%)	786 (12.4%)
AIDS diagnosis, n(%)				
Yes	336 (9.8%)	156 (6.8%)	43 (6.8%)	535 (8.4%)
CD4 count cells/mm3, n(%)				
≤ 300	1156 (33.8%)	580 (25.4%)	172 (27.0%)	1908 (30.1%)
301–500	810 (23.7%)	593 (26.0%)	166 (26.1%)	1569 (24.8%)
≥ 501	1035 (30.2%)	861 (37.8%)	216 (33.9%)	2112 (33.3%)
Unknown	421 (12.3%)	245 (10.8%)	83 (13.0%)	749 (11.8%)
HIV-RNA viral load, n(%)				
≤ 5000	605 (17.7%)	389 (17.1%)	114 (17.9%)	1108 (17.5%)
5001–10,000	208 (6.1%)	172 (7.5%)	48 (7.5%)	428 (6.8%)
10,001–100,000	1222 (35.7%)	922 (40.5%)	231 (36.3%)	2375 (37.5%)
≥ 100,001	1025 (30.0%)	567 (24.9%)	179 (28.1%)	1771 (27.9%)
Unknown	362 (10.6%)	229 (10.0%)	65 (10.2%)	656 (10.4%)
Smoking, n(%)				
No	2201 (64.3%)	924 (40.5%)	188 (29.5%)	3313 (52.3%)
Yes	1092 (31.9%)	1268 (55.6%)	413 (64.8%)	2773 (43.8%)
Unknown	129 (3.8%)	87 (3.8%)	36 (5.7%)	252 (4.0%)
Hepatitis B, n(%)				
Yes	107 (3.1%)	59 (2.6%)	25 (3.9%)	191 (3.0%)
HCV Infection, n(%)				
Negative	2366 (69.1%)	1549 (68.0%)	409 (64.2%)	4324 (68.2%)
Positive	410 (12.0%)	250 (11.0%)	119 (18.7%)	779 (12.3%)
Not tested	646 (18.9%)	480 (21.1%)	109 (17.1%)	1235 (19.5%)
Calendar year, n(%)				
2002–2006	473 (13.8%)	313 (13.7%)	69 (10.8%)	855 (13.5%)
2007–2012	1113 (32.5%)	671 (29.4%)	218 (34.2%)	2002 (31.6%)
2013–2016	1836 (53.7%)	1295 (56.8%)	350 (54.9%)	3481 (54.9%)
Follow-up (months)				
Median (IQR)	26.5 (7.4, 57.1)	23.4 (4.8, 53.5)	25.6 (5.6, 54.8)	25.2 (6.1, 55.6)

### Alcohol consumption and risk of severe liver disease

**Fig. 2:**
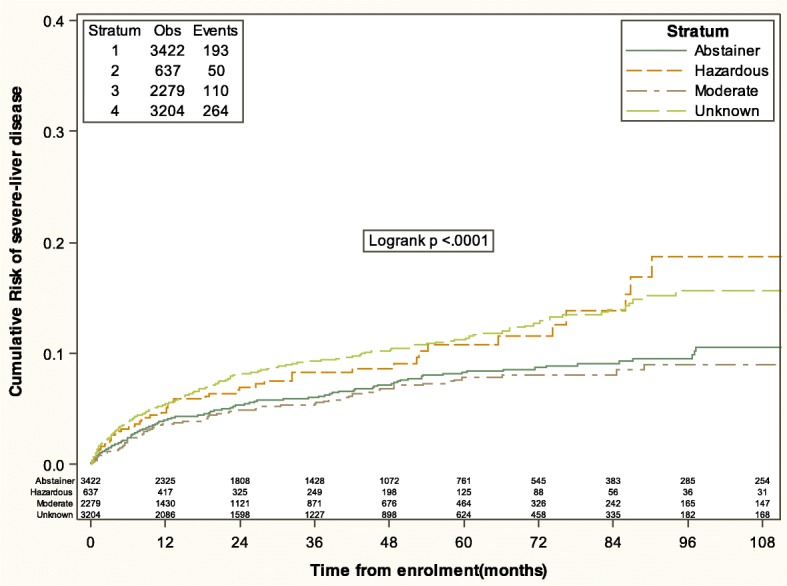
Kaplan Meier estimate of the risk of severe liver disease stratified by alcohol consumption classification

Patients were followed-up for a median [IQR] of 25.2 months [6.1–55.6]. A total of 617 (7%) people experienced the composite SLD outcome (*n* = 506 FIB4 > 3.25, *n* = 110 clinical diagnosis of liver disease, *n* = 1 liver-related mortality). Fig. 2 shows the Kaplan-Meier estimates of the time to SLD according to baseline alcohol consumption level. The estimated cumulative risk of experiencing SLD by 60 months (95% CI) from baseline in abstainers, moderate, hazardous or unknown alcohol consumption were 8.4% (7.1–9.7), 7.9% (6.3–9.5), 10.7% (7.4–14.1) and 11.4% (9.9–12.9), respectively [log-rank *p* < 0.001].

**Fig. 3:**
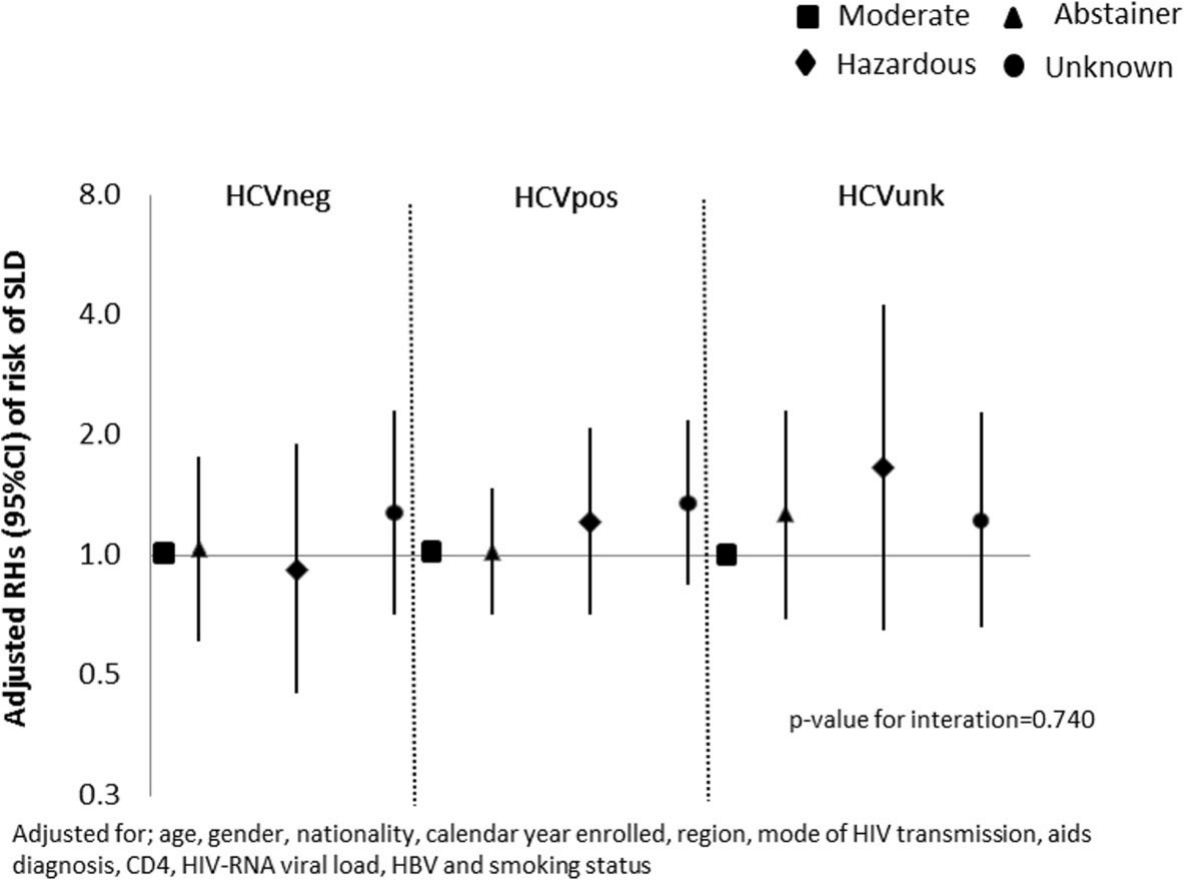
Cox regression adjusted HRs stratified by HCV status for risk of severe liver disease

In univariable Cox regression analyses with moderate drinkers as the comparator group, hazardous drinking and unknown alcohol status were strongly associated with increased risk of SLD (unadjusted HR = 1.61 [95% CI: 1.16–2.26]; *p* = 0.005 and 1.67 [95% CI: 1.34–2.09]; *p* < 0.001 respectively. In contrast, there was no evidence for a difference between abstaining and moderate consumption (unadjusted HR = 1.09 [95% CI: 0.87–1.38]; *p* = 0.446) Table 2**.** After controlling for age, gender, nationality, region, calendar year enrolled, HIV related factors and HBV, and still using the moderate consumption as the comparator, adjusted HR (95%CI) for hazardous drinking and unknown alcohol consumption were [aHR = 1.45 (1.03–2.03; *p* = 0.031) and aHR = 1.37 (1.09–1.72; *p* = 0.007)] respectively. However, after additionally adjusting for mode of HIV transmission and HCV infection status, the effect of hazardous drinking was attenuated (aHR = 1.30 [95% CI: 0.92–1.82]; *p* = 0.129) but unknown alcohol consumption remained associated with risk of SLD (aHR = 1.43 [95% CI: 1.13–1.80]; *p* = 0.003). After further adjustment for smoking status, alcohol consumption was no longer associated with risk of SLD; (aHR = 1.09 [95% CI: 0.86–1.38]; global *p* = 0.446) Table 2. An interaction term between HCV infection and alcohol use was not significant, indicating that the association between level of alcohol consumption and risk of SLD did not differ by HCV status (*p* = 0.740 Fig. 3**).**

**Table 2:** Univariable and multivariable Cox regression analysis for severe liver disease without/with multiple imputation

	Unadjusted RH[95%CI]	*p*-value	global *p*-value	Model 1 RH[95%CI]	*p*-value	global *p*-value	Model 2 RH[95%CI]	*p*-value	global *p*-value	Model 3 RH[95%CI]	*p*-value	global *p*-value	Model 4 RH[95%CI]	*p*-value	global *p*-value
Alcohol consumption															
Abstainer	1.09 (0.87, 1.38)	0.446	<.001	1.08 (0.85, 1.37)	0.506	<.001	1.06 (0.84, 1.34)	0.640	0.007	1.10 (0.87, 1.40)	0.413	0.009	1.09 (0.86, 1.38)	0.498	0.446
Moderate	1.00			1.00			1.00			1.00			1.00		
Hazardous	1.61 (1.16, 2.26)	0.005		1.45 (1.04, 2.04)	0.028		1.45 (1.03, 2.03)	0.031		1.30 (0.95, 1.86)	0.129		1.32 (0.94, 1.85)	0.107	
Unknown	1.67 (1.34, 2.09)	<.001		1.56 (1.24, 1.95)	<.001		1.37 (1.09, 1.72)	0.007		1.43 (1.13, 1.80)	0.003		1.12 (0..86, 1.46)	0.408	
Gender															
Male vs Female	1.17 (0.96, 1.43)	0.117	0.117	1.12 (0.91, 1.38)	0.286	0.286	1.13 (0.92, 1.39)	0.259	0.259	1.15 (0.92, 1.43)	0.213	0.213	1.15 (0.92, 1.43)	0.217	0.217
Age, years															
per 10 years older	1.60 (1.50, 1.71)	<.001	<.001	1.56 (1.45, 1.67)	<.001	<.001	1.48 (1.37, 1.59)	<.001	<.001	1.47 (1.36, 1.59)	<.001	<.001	1.46 (1.34, 1.58)	<.001	<.001
Nationality															
Italian vs Non-Italian	1.75 (1.36, 2.25)	<.001	<.001	1.26 (0.97, 1.65)	0.081	0.081	1.20 (0.92, 1.56)	0.184	0.184	0.97 (0.74, 1.27)	0.829	0.829	0.98 (0.74, 1.28)	0.877	0.877
Geographical Region															
North	1.00		0.096	1.00		0.341	1.00		0.242	1.00		0.391	1.00		0.353
Center	0.83 (0.69, 0.98)	0.031		0.90 (0.75, 1.07)	0.226		0.88 (0.74, 1.05)	0.157		0.94 (0.79, 1.13)	0.553		0.95 (0.79, 1.14)	0.596	
South	0.95 (0.72, 1.27)	0.739		1.08 (0.81, 1.44)	0.601		1.09 (0.81, 1.46)	0.584		1.16 (0.87, 1.58)	0.291		1.19 (0.89, 1.60)	0.240	
Calendar year enrolled															
2002–2006	1.00		<.001	1.00		<.001	1.00		<.001	1.00		<.001	1.00		<.001
2007–2012	0.57 (0.45, 0.71)	<.001		0.52 (0.41, 0.66)	<.001		0.51 (0.40, 0.65)	<.001		0.63 (0.49, 0.79)	<.001		0.59 (0.46, 0.75)	<.001	
2012–2016	1.08 (0.86, 1.35)	0.511		0.88 (0.70, 1.11)	0.269		0.67 (0.53, 0.86)	0.001		0.69 (0.54, 0.89)	0.005		0.64 (0.49, 0.82)	<.001	
AIDS Diagnosis															
Yes vs, No	1.64 (1.30, 2.07)	<.001	<.001	–			1.34 (1.05, 1.73)	0.021	0.021	1.40 (1.09, 1.79)	0.008	0.008	1.40 (1.09, 1.80)	0.008	0.008
CD4 count															
≤ 300	1.00		<.001	–			1.00		<.001	1.00		<.001	1.00		<.001
301–500	0.69 (0.54, 0.90)	0.005		–			0.86 (0.65, 1.13)	0.280		0.91 (0.68, 1.19)	0.487		0.91 (0.69, 1.19)	0.491	
> 500	0.96 (0.77, 1.20)	0.724		–			1.22 (0.95, 1.56)	0.113		1.39 (1.07, 1.78)	0.010		1.39 (1.08, 1.80)	0.008	
Unknown	3.36 (2.72, 4.16)	<.001		–			4.93 (3.60, 6.75)	<.001		2.62 (1.86, 3.70)	<.001		2.43 (1.72, 3.44)	<.001	
Viral load, copies/mL															
≤5000	1.00		<.001	–			1.00		<.001	1.00		0.012	1.00		0.010
5000–10,000	0.64 (0.44, 0.93)	0.018		–			1.09 (0.73, 1.62)	0.670		1.53 (1.02, 2.30)	0.041		1.55 (1.03, 2.33)	0.034	
10,000–100,000	0.63 (0.50, 0.79)	<.001		–			1.12 (0.86, 1.45)	0.410		1.57 (1.19, 2.07)	0.001		1.59 (1.21, 2.10)	0.001	
> 100,000	0.71 (0.56, 0.90)	0.005		–			1.08 (0.82, 1.43)	0.566		1.63 (1.21, 2.19)	0.001		1.64 (1.22, 2.22)	0.001	
Unknown	1.43 (1.12, 1.83)	0.005		–			0.57 (0.44, 0.76)	<.001		1.05 (0.79, 1.38)	0.752		1.06 (0.80, 1.40)	0.652	
HBV infection															
Yes vs, No	1.91 (1.33, 2.74)	<.001	<.001	–			1.92 (1.34, 2.77)	<.001	<.001	1.97 (1.37, 2.84)	<.001	<.001	2.03 (1.41, 2.93)	<.001	<.001
Mode of HIV Transmission															
PWID	1.00		<.001	–			–			1.00		<.001	1.00		<.001
MSM	0.15 (0.13, 0.19)	<.001		–			–			0.45 (0.34, 0.59)	<.001		0.44 (0.33, 0.58)	<.001	
Heterosexual contacts	0.18 (0.15, 0.22)	<.001		–			–			0.44 (0.33, 0.58)	<.001		0.43 (0.32, 0.57)	<.001	
Other	0.32 (0.24, 0.43)	<.001		–			–			0.64 (0.47, 0.89)	0.008		0.62 (0.45, 0.86)	0.004	
HCV infection															
No	1.00		<.001	–			–			1.00		<.001	1.00		<.001
Yes	7.10 (5.89, 8.55)	<.001		–			–			2.91 (2.22, 3.82)	<.001		2.94 (2.24, 3.86)	<.001	
Unknown	1.67 (1.37, 2.05)	<.001		–			–			1.36 (1.10, 1.69)	0.005		1.34 (1.08, 1.67)	0.007	
Smoking status															
No	1.00		<.001	–			–			–			1.00		0.001
Yes	1.39 (1.13, 1.70)	0.001		–			–			–			0.91 (0.73, 1.13)	0.405	
Unknown	2.30 (1.89, 2.80)	<.001		–			–			–			1.46 (1.12, 1.91)	0.006	
With Multiple Imputation															
Alcohol consumption															
Abstainer	1.15 (0.90, 1.47)	0.261		1.13 (0.88, 1.45)	0.339		1.11 (0.87, 1.42)	0.394		1.13 (0.89, 1.44)	0.315		1.13 (0.88, 1.45)	0.353	0.724
Moderate	1.00			1.00			1.00			1.00			1.00		
Hazardous	1.70 (1.24, 2.34)	0.002		1.48 (1.07, 2.05)	0.020		1.43 (1.03, 1.99)	0.037		1.29 (0.93, 1.78)	0.120		1.30 (0.94, 1.81)	0.114	

1. Age, gender, ethnicity, geographical region, calendar year enrolled; 2. Model 1 + HIV-related factors + HBV; 3. Model 2 + Mode of HIV transmission and HCV infection; 4. Model 3 + Smoking status

In the univariable Cox regression analyses, after combining MI estimates from separate multivariable models, results were similar to those in the main analysis. Hazardous drinking was associated with the risk of SLD (unadjusted HR = 1.70 [95% CI: 1.24–2.34]; *p* = 0.002 and abstaining was not associated with risk SLD (unadjusted HR = 1.15 [95% CI: 0.90–1.47]; *p* = 0.261] Table 2. However, as in the main analysis, after adjusting for potential confounders including mode of HIV transmission and HCV infection hazardous drinking was no longer associated with risk of SLD (aHR = 1.29 [95% CI: 0.93–1.78]; *p* = 0.120). Further adjustment for smoking status, alcohol consumption was not associated with risk of SLD (aHR = 1.13 [95%CI: 0.88–1.45]; global *p* = 0.724) Table 2.

## Discussion

One of the objectives of this analysis was to classify drinking behaviour using data on alcohol consumption routinely collected through physician assessment in our cohorts of PLWHIV seen for routine care in Italy. In this analysis involving 9542 PLWHIV, using the NIFN guidelines of whom 6338 (66%) had data on alcohol consumption. In our study population, 10% of individuals were classified as hazardous drinkers, 36% as moderate drinkers and 54% as abstainers. The overall estimate of the current prevalence of alcohol consumption was 46%. Other HIV studies in which alcohol consumption was measured with similar questionnaires have reported prevalence of current use ranging between [27–95%] [18, 21–25]. Our estimate of hazardous use of 10% was similar to reported estimates in other studies ranging from [8–12%] [5, 16, 18, 23, 35]. Other studies using different assessment tools reported higher estimates of alcohol consumption. For example, *Samet J* et al assessed alcohol consumption in HIV positive individuals in USA by means of patient’s interviews using a series of questions on quantity and frequency and also carried out breath alcohol level test [1]. These authors reported 27% with moderate drinking behaviour and 16% with risky drinking. In addition the use of different measurement tools is a further reason to explain lower prevalence of hazardous drinking in our study could be due to collection of alcohol consumption via face to face rather than anonymously.

Thirty four percent of individuals in this analysis had missing data on alcohol consumption. This large proportion of missing data highlights the challenges of collecting complete data on alcohol consumption in observational studies of PLWHIV. It is also unclear whether unreported alcohol consumption may have arisen from questions not being asked by the physician or the participant being unwilling to give information for other unknown reasons. The prevalence of people with missing information was generally consistent with that seen in other HIV studies showing estimates of under-reporting ranging between [7–41%] [8, 25, 36, 37]. Possible reasons for under-reporting of alcohol use include social desirability and fear of the impact on antiretroviral therapy initiation [27, 35, 36]. Of note, it is part of the Italian culture to assume that a drink with a meal is normal consumption which might also explain the under-reporting. Some studies have assessed the extent of under-reporting by comparing self-reported alcohol consumption with blood tests or biomarkers, or interviews carried out by professionals and found a lack of agreement between these measures [27, 36, 38]. Physician interview like ours are likely to measure alcohol consumption even less accurately than self-administered questionnaires [29].

Different classifications of alcohol consumption were used across studies making it difficult to make valid comparisons. It has to be noted that most of other published studies were enriched with people in specific risk groups who are more likely to have alcohol problems [8, 36, 39]. In contrast, our estimates are from a heterogeneous cohort with 12% PWID, 42% MSMs and 40% who acquired HIV through heterosexual contacts. Indeed, we did find that PWID were more likely to have missing data for alcohol consumption which in turn was associated with higher risk of SLD.

This analysis also set out to investigate whether our measure of alcohol consumption was useful to predict the risk of SLD. Seven percent of our study population experienced SLD over follow-up. Although the risk of SLD appeared marginally lower for moderate drinkers compared with abstainers, this was not statistically significant. A lower risk in moderate drinkers compared to abstainers has been previously documented and a possible explanation could be due to the fact that patients who are currently abstaining may include individuals who were never drinkers as well as those who previously drank and had to abstain due to medical reasons or other reasons [40]. In our study it was not possibly to separate these groups. We found an association of hazardous drinking with increased risk of SLD that was largely independent of baseline demographic and HIV related factors and HBV. However, after further adjusting for mode of HIV transmission, HCV infection and smoking status the association was largely attenuated. *Lim JK* et al, in 2111 PLWHIV also found a moderate increased risk of advanced fibrosis in people with hazardous alcohol consumption (aOR = 1.26 (95%CI: 0.87–1.82) compared to non-hazardous drinkers) after adjusting for a number of potential confounders including HCV infection [11]. In another study including 308 PLWHIV, in which heavy alcohol consumption was defined as > 2 drinks/day or ≥ 5 drinks per occasion and > 1 drink per day or ≥ 4 drinks per occasion for men and women respectively, reported that 10% of the study population developed of liver fibrosis. Consistent with our results, the authors found a moderate difference in risk according to alcohol consumption and no significant association between heavy alcohol consumption and risk of advanced liver fibrosis (aOR = 1.14 [95%CI: 0.47–2.77] compared to non-heavy alcohol consumption; *p* = 0.77) [41]. In contrast, Chaudhry et al. 2009 did find an association between alcohol consumption and liver fibrosis measured using the APRI score after adjusting for potential confounders including HCV infection[aRR = 2.30 (95%CI: 1.26–4.17)]. Of interest, also in their analysis there was no evidence that the association between alcohol consumption and the risk of SLD varied by HCV infection status.

Our study has several other limitations that should be addressed. First, we used a time-fixed covariate at enrolment to classify individuals’ drinking behaviour for the study period, although it is possible that drinking habits changed over follow-up potentially leading to a dilution of the association. This was done mainly to simplify the analysis as mechanisms of time-dependent confounding in this context are largely unexplored and potentially difficult to address by means of a standard Cox regression analysis. Secondly, as typical in the observational setting, we have to assume that results cannot be explained by residual or unmeasured confounding. In addition, because of the large proportion of people with missing data, selection bias cannot be entirely ruled out. People lacking information on alcohol were indeed different from those with complete data for a number of factors known to be associated with the risk of SLD (Additional file 2: Table S2). However, use of multiple imputation gave similar results to the main analysis and the amount of missing data observed in out cohorts is consistent with other HIV cohorts collecting data on alcohol use. Thirdly, data collected on mode of HIV transmission in ICONA and HepaICONA is not able to distinguish between ex-PWID and current PWID, leading to potential residual confounding due to misclassification. Finally, it’s possible that the physicians may not ask the questions on alcohol use in standardised fashion in accordance with the format on the eCRF.

## Conclusion

In conclusion, we evaluated the value of information on alcohol consumption obtained by brief physician interview of PLWHIV to predict their future risk of occurrence of SLD. We found an association between alcohol consumption and risk of SLD which was however partly explained by differences in HCV status, HIV mode of transmision and smoking. There was no evidence that the association was stronger when restricting the analysis to the HCV-infected population. It is conceivable that the weak association found is due to misclassification of the exposure, so efforts should be made in order to collect more accurate information on alcohol consumption in cohorts of HIV/HCV infected individuals. In particular data collection on historical alcohol consumption including items which could allow to distinguish between people who had currently stopped drinking from those who never drank would be useful for future studies.

## Supplementary information

**Additional file 1. Table S1.** List of Ethic Committees that Approved Icona Study.

**Additional file 2. Table S2.** Patients’ characteristics stratified by reported and non-reported alcohol consumption.

## Data Availability

The datasets generated and/or analysed during the current study are not publicly available due [reasons below] but are available from the corresponding author on reasonable request. As a group we are very open to collaboration and in involving other researchers in our work. However, we strongly feel that we cannot make a full dataset publicly available for several reasons: 1. We are extremely concerned about confidentiality – since these patients may be identified by combinations of person-specific characteristics within the database, and the database includes sensitive data such as HIV status, HCV status, as well as data on intravenous drug use as well as sexual preferences. 2. Further, being a multicentre study, the study has gone through a long process of being approved by the individual clinics and national Ethical Committees. Such approvals do not include granting public access to the data being collected. This would mean that we would have to go back for renewed evaluation by all clinics as well as by national Ethical Committees in all sites.

## Abbreviations

ART: Antiretroviral treatment
CRF: Case report form
DAA: Direct acting antivirals
HCV: Hepatitis C virus
HIV: Human immunodeficiency virus
NIFN: National institute for food and nutrition
PLWHIV: People living with HIV
PWID: People who inject drugs
SLD: Severe liver disease
WHO: World health organisation
