# Supplementary material for: Is physician assessment of alcohol consumption useful in predicting risk of severe liver disease among people with HIV and HIV/HCV co-infection?
Source: BMC Public Health. 2019 Oct 15;19:1291. doi: 10.1186/s12889-019-7608-1 (PMC6794785; doi:10.1186/s12889-019-7608-1)
Supplement: Supplementary file 2 — Additional file 2. Table S2. Patients’ characteristics stratified by reported and non-reported alcohol consumption. [file 12889_2019_7608_MOESM2_ESM.docx]

Table S2 Patients’ characteristics stratified by reported and non-reported alcohol consumption

| **Characteristics** | **Reported (N=6338)** | **Non-reported (N=3204)** | ***p-value** |
| --- | --- | --- | --- |
| ***Gender, n(%)*** |  |  | <.001 |
| Male | 4884 (77.1%) | 2584 (80.6%) |  |
| Female | 1454 (22.9%) | 620 (19.4%) |  |
| ***Age, years*** |  |  |  |
| Median (IQR) | 38 (31, 46) | 39 (31, 48) | <.001 |
| ***Mode of HIV Transmission, n(%)*** |  |  | <.001 |
| PWID | 731 (11.5%) | 431 (13.5%) |  |
| Homosexual contacts | 2663 (42.0%) | 1276 (39.8%) |  |
| Heterosexual contacts | 2543 (40.1%) | 1094 (34.1%) |  |
| Other/Unknown | 401 (6.3%) | 403 (12.6%) |  |
| ***Nationality, n(%)*** |  |  | <.001 |
| Italian | 5003 (78.9%) | 2625 (81.9%) |  |
| ***Site geographical position, n(%)*** |  |  | <.001 |
| North | 3103 (49.0%) | 2078 (64.9%) |  |
| Centre | 2449 (38.6%) | 973 (30.4%) |  |
| South | 786 (12.4%) | 153 (4.8%) |  |
| ***AIDS diagnosis, n(%)*** |  |  |  |
| Yes | 535 (8.4%) | 266 (8.3%) | 0.817 |
| ***CD4 count cells/mm^3^, n(%)*** |  |  | <.001 |
| ≤300 | 1908 (30.1%) | 839 (26.2%) |  |
| 301-500 | 1569 (24.8%) | 697 (21.8%) |  |
| ≤501 | 2112 (33.3%) | 884 (27.6%) |  |
| Unknown | 749 (11.8%) | 784 (24.5%) |  |
| ***HIV-RNA viral load, n(%)*** |  |  | <.001 |
| ≤5000 | 1108 (17.5%) | 633 (19.8%) |  |
| 5001-10000 | 428 (6.8%) | 177 (5.5%) |  |
| 10001-100000 | 2375 (37.5%) | 997 (31.1%) |  |
| ≥100001 | 1771 (27.9%) | 744 (23.2%) |  |
| Unknown | 656 (10.4%) | 653 (20.4%) |  |
| ***Smoking, n(%)*** |  |  | <.001 |
| No | 3313 (52.3%) | 424 (13.2%) |  |
| Yes | 2773 (43.8%) | 480 (15.0%) |  |
| Unknown | 252 (4.0%) | 2300 (71.8%) |  |
| ***Hepatitis B, n(%)*** |  |  |  |
| Yes | 191 (3.0%) | 64 (2.0%) | 0.004 |
| ***HCV Infection, n(%)*** |  |  | <.001 |
| Negative | 4324 (68.2%) | 1374 (42.9%) |  |
| Positive | 779 (12.3%) | 439 (13.7%) |  |
| Not tested | 1235 (19.5%) | 1391 (43.4%) |  |
| ***Calender yr enrolled, n(%)*** |  |  | <.001 |
| 2002-2006 | 855 (13.5%) | 299 (9.3%) |  |
| 2007-2012 | 2002 (31.6%) | 929 (29.0%) |  |
| 2013-2016 | 3481 (54.9%) | 1976 (61.7%) |  |
| ***Follow-up (months)*** |  |  |  |
| Median (IQR) | 25.2 (6.1, 55.6) | 23.9 (6.6, 51.8) | 0.002 |

*Kruskall Wallis test chi-squared test
